# Imaging biomarker of beta‐amyloid pathology limits cognitive plasticity in healthy aging: A multi‐center intervention study

**DOI:** 10.1002/alz.093785

**Published:** 2025-01-09

**Authors:** Gérard N Bischof, Tim Fellerhoff, Kathrin Giehl, Alexander Drzezga

**Affiliations:** ^1^ University of Cologne, Faculty of Medicine and University Hospital Cologne, Cologne Germany; ^2^ University of Cologne, University Hospital of Cologne, Department of Nuclear Medicine, Multimodal Neuroimaging Group, Cologne, Germany;Medical Faculty, Heinrich Heine University Düsseldorf, Germany, Cologne Germany; ^3^ Research Center Jülich, Institute for Neuroscience and Medicine ‐ Molecular Organization of the Brain (INM‐2), Jülich Germany; ^4^ University of Cologne, Faculty of Medicine and University Hospital Cologne, Department of Nuclear Medicine, Cologne Germany

## Abstract

**Background:**

Experience‐dependent cognitive plasticity can be induced by cognitive training well into the later decades of life. Although some prerequisites (education, intelligence) and constraints (age, genetic predisposition) on the extent of cognitive plasticity have been discussed, the impact of molecular age‐related changes in the form of fibrillar amyloid pathology on plasticity remains to be investigated. Expanding our knowledge of potential contributors to the observed heterogeneity in intervention benefits may allow us to improve non‐pharmaceutical interventions

**Method:**

Seventy‐six healthy older adults (mean age = 68.5, SD = 5.8) participated in a cognitive intervention study with twelve 60‐minute sessions. Tasks targeted executive function, memory, attention, and processing speed. Neuropsychological tests were conducted at baseline, posttraining, and three months. Outcome measures included training improvement, immediate transfer task improvement, and long‐term transfer maintenance. Positron emission tomography (PET) with 18F‐florbetaben imaged beta‐amyloid plaques. General linear models, controlling for age, education, and intelligence, assessed the influence of global SUVR on intervention outcomes.

**Result:**

We observed significant negative effects of global amyloid on training improvement, controlling for baseline effects, age, education, and intelligence, with individuals with higher SUVRs showing significantly less training improvement. Similarly, significant amyloid deposition was negatively associated with transfer maintenance to follow‐up, again controlling for post‐training, age, education, and intelligence. Interestingly, immediate transfer improvement at follow‐up was not related to amyloid depositio

**Conclusion:**

Our study reveals that amyloid pathology significantly hinders cognitive training benefits in healthy older adults. Amyloid limits cognitive plasticity induction, posing challenges for long‐term maintenance of training effects. The interplay between cognitive resilience and amyloid emerges, emphasizing the need for tailored cognitive interventions considering neuropathological factors. While cognitive training stimulates plasticity, its efficacy is constrained by amyloid presence, emphasizing the necessity of comprehensive strategies for addressing cognitive decline in aging individuals.

